# Translation and Validation of the Turkish Version of the Quality of Postoperative Recovery Score QoR-15: A Multi-Centred Cohort Study

**DOI:** 10.5152/TJAR.2022.21417

**Published:** 2022-12-01

**Authors:** Onur Selvi, Mustafa Azizoğlu, Gülhan Temel, Serkan Tulgar, Ahish Chitneni, Ece Nur Çınar, Zeliha Özer, Yavuz Gürkan

**Affiliations:** 1 Department of Anaesthesiology, Cambridge University Addebrooke’s Hospital, Cambridge, United Kingdom; 2 Department of Anaesthesiology, Mersin University Faculty of Medicine, Mersin, Turkey; 3 Department of Anaesthesiology, Samsun University Faculty of Medicine, Samsun, Turkey; 4 Department of Anaesthesiology, A.T Still University in Arizona (SOMA), Arizona, USA; 5 Department of Anaesthesiology, West Middlesex Hospital, London, United Kingdom; 6 Department of Anaesthesiology, Maltepe University Faculty of Medicine, İstanbul, Turkey; 7 Department of Anaesthesiology, Koç University Faculty of Medicine, İstanbul, Turkey

**Keywords:** ERAS, pain, perioperative care, QoR-15, QoR-40

## Abstract

**Objective:**

The Quality of Recovery-15 questionnaire is a self-rated questionnaire used to assess the quality of the postoperative recovery and health status of patients in the early period following surgery. The aim of this study was to assess the reliability, validity, and responsiveness of the Turkish version of the Quality of Recovery-15.

**Methods:**

After approval by the Maltepe University local ethics committee, this observational study was conducted among patients who received surgical interventions at Mersin University Hospital between July 2019 and January 2020. Reliability, feasibility, and validity were assessed to validate the Turkish version of the Quality of Recovery-15.

**Results:**

The completion rate of the form was determined to be 92% and a total of 200 patients were enrolled in the study. The Cronbach’s alpha of the global Turkish version of the Quality of Recovery-15 was 0.927. Test–retest reliability was 0.84 [CI 95%: 0.75-0.90] and Cohen’s effect size was 0.319. The total standardized response mean was determined as 0.53.

**Conclusions:**

This is the first study in which the Quality of Recovery-15 scale was translated into Turkish with our knowledge. The Turkish version of the Quality of Recovery-15 showed satisfactory reliability and validity in evaluating the quality of recovery after surgery in the Turkish population.

**Main Points**

- This study evaluated the Turkish version of a questionnaire that is used for the assessment of quality of recovery after surgery, the Quality of Recovery-15.
- The study proves that the scale could be administered in Turkish language to Turkish population.
- Its current form can easily be used in quality improvement studies in healthcare institutions.

## Introduction

The quality of the recovery period after anaesthesia and the level of patient satisfaction during the postoperative period continue to be an essential topic of discussion. Several scales and methods have been developed to evaluate the quality of recovery.^1-3^ Classically, to evaluate the recovery process postoperatively, factors such as comparison of recovery times after anaesthesia and surgery, nausea, vomiting, level of pain, and complication rates are evaluated.^1^ Although these topics are fundamental, they do not address the recovery process from the patient’s point of view. However, with the widespread use of minimally invasive interventions to reduce postoperative mortality and morbidity, the implementation of the Enhanced Recovery After Surgery protocols increased the overall interest in the postoperative recovery period and the importance of evaluating outcomes. In addition to existing conventional scales that measure classical parameters, new tools prioritising patients’ satisfaction have been developed to meet the arising needs in this field. One such tool is the Quality of Recovery-15 (QoR-15) scale.^2^

The QoR-40 recovery scale, which was previously prepared by Karaman et al^3^, has been widely used and validated in many languages. In 2013, a shorter 15-item form was created based on the QoR-40. The QoR-15 scale contains similar psychometric features compared to the QoR-40, but it is a more straightforward form to apply. Many previous studies have shown that this new scale can be used as effectively as QoR-40.^4^ This scale, which is a simpler and more manageable version of the previous recovery scale QoR-40, has neither been translated nor validated in Turkish. This study aims to translate and validate the QoR-15 scale in the Turkish language (QoR-15T). 

## Methods

After approval by the Maltepe University Medical Faculty local ethics committee on 26 June 2019 with the registration number 2019/900, this multicentred observational study was conducted among patients who received surgical interventions at Mersin University Hospital between July 2019 and January 2020. Turkish translation and adaptation of the QoR-15 were completed at Maltepe University Hospital. All patients older than 18 years old, undergoing elective surgery, and can understand and read the Turkish language were invited to the study. During the patients’ pre-anaesthetic visit, comprehensive information regarding the study and the purpose of the study was conveyed to the patient. All patients accepted to participate in the study during their pre-anaesthetic visit, and information packets were given to all participants. Patients with a medical history that impairs comprehension and communication skills, who do not speak Turkish, who have a history of alcohol or substance use, and who are under 18 and over 80 years old have been excluded from participation in the study. 

Demographic data, American Society of Anaesthesiologists (ASA) scores, and duration and type of surgery were obtained from anaesthesia records, while preoperative and postoperative QoR-15 scores were collected from the forms filled out by patients. Surgery type was classified as minor, major, and intermediate surgery based on the Danish and Swedish versions of the study.^5,6^ The length of stay was calculated from the patient records as the time between the end of surgery and the time patients were discharged. In addition, the status of the existence of any complications in the first 24 hours postoperatively was recorded such as hypoxia, hypotension, and bleeding.

It was ensured that the patients consenting and participating in the study completed the preoperative QoR-15TR questionnaire in the ward during the preoperative period. Patients who did not complete the form or could not be reached during the follow-up period were also excluded from the study.

All patients were instructed to fill out the questionnaire on the first day after surgery. 

### Translation and Creation of the Turkish Form

Translation and creation of the Turkish form were completed at Maltepe University Hospital with permission from the authors of the original study.^7^ The World Health Organization’s “Process of translation and adaptation of instruments” was used to create the Turkish QoR-15 form.^5,6^ Initially, it was translated from English to Turkish by 2 bilingual translators who used Turkish and English as their mother tongue before the commencement of the project. These 2 translations were combined to form the basis of the Turkish QR-15 form. Later, this translated form was translated back into English by 2 independent translators. Finally, 4 translators collaborated on the proforma, and the latest version they agreed upon was tested among 10 randomly chosen participants to appraise the comprehensibility of the questions. Thus, the final shape of the form was created.

The patients to be included in the study were determined as the patients to be operated on 3 random days of a week. The study was terminated after the return of 200 complete forms meeting the inclusion criteria.

Approximately 150 patients were included in the previous Swedish and Danish QoR-15 validation studies, with 10 patients per question.^1,2^ Although QoR-15 took sample sizes in the Swedish and Danish validation studies, we included 200 patients in our study considering possible data loss.

### Statistical Analysis

All patients’ information regarding age, ASA score, gender, name, grade (minor, intermediate, major), duration of surgery, if any, and postoperative complications were recorded. The preoperative and postoperative responses to QoR-15TR were determined (Table 1). Distribution was analysed with the Shapiro–Wilk test. Relationships between categorical variables were tested using the chi-square test, and mean differences between groups were tested using analysis of variance. Descriptive statistics for quantitative data were shown as mean ± standard deviation (SD) or mean value and qualitative data were presented in percentage and frequency. The associations were measured using the intra-class correlation coefficient, while internal consistency was determined by Cronbach’s alpha. Cohen’s effect size was calculated as the average change in QoR-15D score (from preoperative to postoperative) divided by the SD at baseline (preoperative). The standardised response mean was calculated by dividing the mean change in the QoR-15TR value by this SD value. All analysis was performed with International Business Machines Statistical Package for the Social Sciences Statistics for Macintosh, version 22.0 (IBM Corp, Armonk, NY, USA). 

The floor and ceiling effects of the postoperative QoR-15TR scores and the skewness of the distributions were measured. The presence of the ceiling and floor effect was formulated as the existence of more than 15% of the participants scoring a score of the highest or lowest possible values.

The construct validity of the QoR-15TR score was compared with the results of tests measuring similar properties. Reliability was determined with Cronbach’s alpha, split-half reliability, while test–retest reliability was determined with intra-class correlation coefficient. Responsiveness was determined by Cohen’s effect size and standardized response mean.

## Results

**Figure 1.**
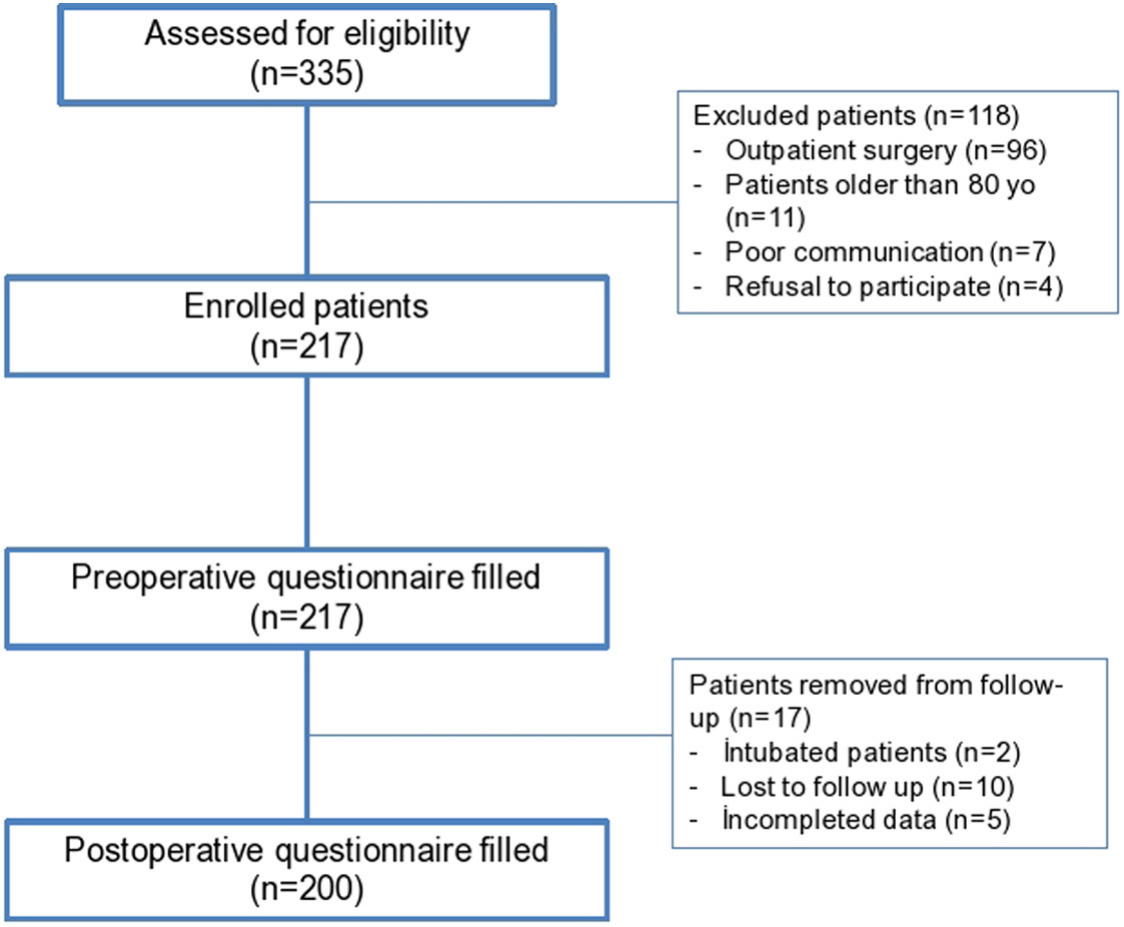
Flowchart.

A total of 335 patients were evaluated for eligibility for the study. Of these patients, 217 patients who met the inclusion criteria were included in the study. Two of these patients were excluded from the study because they were followed up in intensive care under mechanical ventilation support within the first 24 hours postoperatively. Ten patients did not return their postoperative questionnaire forms, and it was observed that 2 patients filled out their forms incompletely (Figure 1). For this reason, the completion rate of the form was determined to be 92%. Demographic information and clinical information of the patients are given in Table 1. The patients were grouped as minor, intermediate, and major surgeries according to the content of their surgery. The characteristics of each group are given in Table 2.

There was no significant difference between the surgical groups in terms of gender and age distribution (*P* = .11). There was a significant difference between the groups in terms of ASA scores (*P* < .001). A significant difference was observed between the groups (*P* < .001) in terms of length of hospital stay and complications in the first 24 hours. When the preoperative QoR-15TR score and postoperative QoR-15TR scores were examined, it was observed that there was no difference between the 3 surgical groups (*P* = .20 and *P* = .87). The median value of the postoperative QoR-15TR score was 118 ± 28, 116 ± 27, and 115 ± 10 for minor, intermediate, and major surgeries, respectively, according to the surgery groups.

There was no significant difference between the postoperative QoR-15TR scores (116.79 ± 27.83) of the female and the postoperative QoR-15TR scores of the male patients (119.13 ± 26.83) in all surgical groups (*P* = .40, *P* = .55).

Cronbach’s alpha value for postoperative QoR-15TR scores was 0.927 and the corrected item-total correlation is shown in Table 3. Test–retest reliability was 0.84 [CI 95%: 0.75-0.90] and Cohen’s effect size was 0.319. There was no significant difference between preoperative QoR-15TR scores (121 ± 24) and postoperative QoR-15TR scores (118 ± 27) with a *P* >.05. Cohen’s effect size was 0.319 and the standardized response mean (SRM) was determined as 0.53. Reliability and responsiveness for each question in the QoR-15TR are shown in Table 3.

Considering the intra-class correlation coefficient, while there was a negative correlation between the duration of surgery and the postoperative QoR-15TR scores (−0.262, *P* < .001), a negative correlation was found with the postoperative length of stay (−0.257, *P* < .001). (Table 4)

While the postoperative QoR-15TR score distribution did not show ceiling and floor effects, the curve sloped to the right and its values were between 44 and 150.

## Discussion

As a result of this study, it was determined that the Turkish version of the QoR-15 test was a valid, reliable, and responsive test, and it was found that this test could be used for the evaluation of postoperative recovery in the Turkish population. The fact that the clinical feasibility of the QoR-15TR is above the recommended values indicates that this test can be successfully used in clinical practice.

The “content validity” of the test has been previously tested and validated.^7^ However, criterion validity was not evaluated against a gold standard measurement tool for postoperative recovery, which does not exist in clinical practice.

Construct validity of QoR-15TR was verified as at least 75% of the results are in accordance with the hypotheses obtained in the sample study.^8^ There was a negative correlation, as expected, between the length of hospital stay and the duration of the surgery. There was no correlation between age and the results of QOR-15TR scores. Although there was a difference between the groups in terms of ASA scores and the frequency of complications, there was no difference in terms of postoperative QoR-15TR scores. This information proposes that an adequate and proper administration of successful multimodal analgesic treatment and careful patient care may have improved postoperative QoR-15 scores in all types of surgical groups. These outcomes all support the construct validity of the QoR-15TR, and consistency between the original study and the Turkish version was determined.^7^

The QoR-15TR showed a high level of internal consistency and the Cronbach’s alpha value was determined to be 0.92, a value in the range of suggested 0.70-0.90.^9^ The fact that the item−total score correlation coefficients of the scale are between 0.460 and 0.780 indicates that the questions are relevant and related to the subject being studied.

The test–retest reliability value, which was checked for the reliability of the scale, was found to be 0.84 in total. This value is between the recommended value of 0.76-0.98.^10^ The data show that the reliability of the QoR-15 TR scale is highly confirmed. “Responsiveness,” seen as a feature of a measuring device, is an indicator showing the sensitivity of the method used over time. The SRM value used for responsiveness is calculated by dividing the change in mean value by standard deviation.^11^ Cohen’s effect size and SRM values state that the QoR-15TR shows superior responsiveness than the original study. Combined with the absence of floor and ceiling effects, the QoR-15TR scale is practically capable of detecting clinical changes to be investigated in the evaluation of the quality of recovery.^12^

Considering the inclusion rate in the study, 200 of 217 patients returned the forms and were included in the study. In this case, the participation rate was 92%, and this value is close to sample studies and higher than the expected value for survey studies.^13^ Being able to answer the questions with a numerical scale between 0 and 10 may have increased the comprehensibility and utility of the test and enabled more participants to fill the test correctly. 

Failure to understand the questions by the participants and mixed test methods and contents may cause erroneous results in patient-based assessment scales, and generalization of the outcome to larger groups becomes impossible. For this reason, a high participation rate prevents inaccurate results and increases the credibility of the test results.^14^

Overall pain score or opioid consumption is traditionally assessed based on the quality of the postoperative period, which provides limited information for investigators. Therefore, a multidimensional recovery scale, such as the QoR-15, has a higher clinical value. This scale, which has become a promising tool for the assessment of the quality of the recovery period, questions the various aspects of recovery in 5 different areas: pain, physical comfort, physical independence, psychological support, and emotional state. Thus, it is a scale that can be used both in clinical studies and for comparing the results of new treatment strategies.^15^ For these reasons, the use of the QoR-15 scale was recommended by the “Standardized Endpoints in Perioperative Medicine” initiative and the European Society of Anaesthesia in clinical studies investigating post-surgical patient comfort and pain level.^16^

A limitation of our study is that the time to complete the test is not measured. In the original research, it was stated that the completion of QoR-15 was less than 3 minutes.^5^ It can be predicted that this scale, which is derived from the QR-40 test used in the evaluation of the review and previously translated into Turkish, can be answered in a shorter time.

## Conclusion

As a result, this is the first study in which the QoR-15 scale was translated into Turkish with our knowledge and it is a reliable, valid, highly responsive, and clinically viable tool that questions the quality of the postoperative recovery process in Turkish. We believe the widespread use of the Turkish version of the scale may contribute to the transformation of recovery care into a patient-oriented service.

## Figures and Tables

**Table 1.** Patient Demographic and Surgical Characteristics

Female (n)	113 (56.5%)
Male (n)	87 (43.5%)
Age (years)	47.09 ± 15.51
**ASA physical status**	
ASA I (n)	54 (27.0%)
ASA II (n)	110 (55.0%)
ASA III (n)	36 (18.0%)
Duration of surgery (minutes)	88.74 ± 78.86 (min-max)
Duration of postoperative stay (day)	1.65 ± 1.222
**Extent of surgery**	
Minor (n)	124 (62.0%)
Intermediate (n)	63 (31.5%)
Major (n)	13 (6.5%)
**Postoperative complications in first 24 hours**	
Yes (n)	30 (15%)
No (n)	170 (85%)
Preoperative QoR-15TR score	121.28 ± 23.90
Postoperative QoR-15TR score	117.80 ± 27.36

The data presented as mean ± standard deviation (range), median (interquartile range), or frequencies (percentage).

ASA, American Society of Anaesthesiologist; QoR-15TR, Turkish version of the Quality of Recovery-15.

**Table 2.** Patient Characteristics According to Extent of Surgery

Variable	Minor	Intermediate	Major	*P*
Female	63 (50.8%)	41 (65.1%)	9 (69.2%)	
Male	61( 49.2%)	22 (34.9%)	4 (30.8%)	.112
Age	46.32 ± 16.371	46.78 ± 14.15	55.92 ± 10.981	.103
ASA I	46 (37.1%)	8 (12.7%)	0 (0.0%)	
ASA II	62 (50.0%)	40 (63.5%)	8 (61.5%)	<.001*
ASA III	16 (12.9%)	15 (23.8%)	5 (38.5%)	
Duration of surgery (minutes)	67.97 ± 44.61	93.63 ± 67.05	263.08 ± 148.29	<.001*
Duration of postoperative stay (day)	1.31 ± 0.71	1.75 ± 0.98	4.46 ± 2.18	<.001*
Postoperative complications within 24 hours after surgery				
Yes	11	14	5	
No	113	49	8	.03*
Preoperative QoR-15TR score	119.94 ± 25.93	121.62 ± 20.76	132.38 ± 14.35	.201
Postoperative QoR-15TR score	118.56 ± 28.68	116.73 ± 27.32	115.77 ± 10.42	.877

Results are presented as mean ± standard deviation (range), median (interquartile range), or frequencies (percentage). Minor surgery: therapeutic hysteroscopic procedures, vaginal surgery for prolapse or incontinence, minor orthopaedic surgeries, and surgery of peripheral nerves. Intermediate surgery: diagnostic laparoscopies, laparoscopic or open hernia repair, laparoscopic cholecystectomies, laparoscopic sterilization, laparoscopic surgery for endometriosis, laparoscopic or open salpingo-oophorectomies, therapeutic arthroscopies, surgery of the extremities, surgery of the elbow, shoulder, or knee. Major surgery: colorectal surgery, hysterectomies, and major orthopaedic surgeries.

ASA, American Society of Anaesthesiologists; QoR-15TR, Turkish version of Quality of Recovery-15.

**Table 3.** Mean, Change, Responsiveness, Test–Retest Reliability, and Corrected Term of the Postoperative QoR-15TR

	Mean ± SD	Change From Baseline ± SD	PreQoR SD	PostQoR SD	Cohen’s Effect Size	SRM	Test–Retest	Corrected Item—Total Correlation
1	9.74 ± 0.65	0.00 ± 0.00	9.743 ± 0.657	9.743 ± 0.657	0.000	0.00	1.0 [(−1,0)-(−1,0)]	0.420
2	9.48 ± 0.88	0.11 ± 0.67	9.543 ± 0.852	9.429 ± 1.037	0.134	−0.16	0.74 [0.55-0.85]	0.658
3	9.28 ± 0.96	0.34 ± 0.99	9.457 ± 0.780	9.114 ± 1.323	0.440	−0.34	0.54 [0.33-0.70]	0.541
4	9.51 ± 0.97	0.11 ± 0.52	9.571 ± 0.815	9.457 ± 1.172	0.140	−0.21	0.85 [0.77-0.91]	0.547
5	9.60 ± 0.91	0.11 ± 0.47	9.657 ± 0.802	9.543 ± 1.067	0.142	−0.24	0.86 [0.78-0.92]	0.629
6	9.31 ± 1.02	0.05 ± 0.23	9.343 ± 0.998	9.286 ± 1.073	0.057	−0.24	0.97 [0.94-0.98]	0.788
7	8.92 ± 1.17	−0.02 ± 0.92	8.914 ± 1.245	8.943 ± 1.282	0.023	0.03	0.73 [0.53-0.85]	0.494
8	9.67 ± 0.94	0.14 ± 1.24	9.743 ± 0.817	9.600 ± 1.376	0.175	−0.11	0.39 [0.12-0.60]	0.623
9	9.34 ± 0.88	−0.11 ± 0.71	9.286 ± 1.100	9.400 ± 0.775	0.104	0.15	0.70 [0.52-0.82]	0.662
10	9.12 ± 1.15	−0.02 ± 0.66	9.114 ± 1.207	9.143 ± 1.192	0.024	0.04	0.84 [0.71-0.91]	0.646
11	9.10 ± 1.12	0.25 ± 1.06	9.229 ± 1.060	8.971 ± 1.403	0.243	−0.24	0.61 [0.38-0.77]	0.328
12	9.45 ± 0.98	0.17 ± 0.74	9.543 ± 0.950	9.371 ± 1.140	0.180	−0.22	0.73 [0.54-0.85]	0.376
13	9.10 ± 0.77	0.54 ± 0.85	9.371 ± 0.770	8.829 ± 0.985	0.705	−0.63	0.44 [0.20-0.64]	0.046
14	8.78 ± 1.13	0.25 ± 0.88	8.914 ± 1.222	8.657 ± 1.211	0.210	−0.29	0.71 [0.51-0.84]	0.240
15	8.67 ± 1.11	0.37 ± 1.00	8.857 ± 1.264	8.486 ± 1.173	0.294	−0.37	0.63 [0.39-0.78]	0.537
Total	139.07 ± 8.07	2.20 ± 4.13	140.171 ± 6.905	137.971 ± 9.550	0.319	−0.53	0.84 [0.75-0.90]	NA

Test–retest are presented as intra-class correlation coefficient and 95% CI.

Test−retest: 0.84 [CI 95%: 0.75-0.90], Cohen’s effect size: 0.319.

QoR-15TR, Turkish version of the QoR-15; SD, standard deviation; SRM, Standardized Response Mean.

**Table 4.** Correlations—Intra-Class Correlation Coefficient

		Preop-QoR-15	Postop-QoR-15
Duration of surgery	Correlation coefficient	−0.031	−0.262
	*P*	.664	<.001
Postoperative stay	Correlation coefficient	−0.27	−0.257
	*P*	.705	<.001
Age	Correlation coefficient	-	0.1
	*P*	-	.568

Preop-QoR-15, pre-operative Quality of Recovery-15 scale; postop-QoR-15, post-operative Quality of Recovery-15 scale.
